# Interpersonal Distance During Real-Time Social Interaction: Insights From Subjective Experience, Behavior, and Physiology

**DOI:** 10.3389/fpsyt.2020.00561

**Published:** 2020-06-12

**Authors:** Leon O. H. Kroczek, Michael Pfaller, Bastian Lange, Mathias Müller, Andreas Mühlberger

**Affiliations:** ^1^Department of Psychology, Clinical Psychology and Psychotherapy, University of Regensburg, Regensburg, Germany; ^2^Department of Research and Development, VTplus GmbH, Würzburg, Germany

**Keywords:** Virtual Reality, psychophysiology, social anxiety, approach, avoidance

## Abstract

Physical distance is a prominent feature in face-to-face social interactions and allows regulating social encounters. Close interpersonal distance (IPD) increases emotional responses during interaction and has been related to avoidance behavior in social anxiety. However, a systematic investigation of the effects of IPD on subjective experience combined with measures of physiological arousal and behavioral responses during real-time social interaction has been missing. Virtual Reality allows for a controlled manipulation of IPD while maintaining naturalistic social encounters. The present study investigates IPD in social interaction using a novel paradigm in Virtual Reality. Thirty-six participants approached virtual agents and engaged in short interactions. IPD was varied between 3.5 and 1 m by manipulating the distance at which agents reacted to the participant's approach. Closer distances were rated as more arousing, less pleasant, and less natural than longer distances and this effect was significantly modulated by social anxiety scores. Skin conductance responses were also increased at short distances compared to longer distances. Finally, an interaction of IPD and social anxiety was observed for avoidance behavior, measured as participants' backward motion during interaction, with stronger avoidance related to close distances and high values of social anxiety. These results highlight the influence of IPD on experience, physiological response, and behavior during social interaction. The interaction of social anxiety and IPD suggests including the manipulation of IPD in behavioral tests in Virtual Reality as a promising tool for the treatment of social anxiety disorder.

## Introduction

Interpersonal distance (IPD), the physical space between persons, sets the ground for social interactions. As a part of non-verbal communication, IPD allows to coordinate social behavior in face-to-face encounters (1). IPD reflects the feeling of comfort in social situations and is largely dependent on relational and cultural factors as well as positive or negative attitudes (2, 3). Different zones of spatial distances have been related to different social functions (4): Intimate space (0–45 cm), personal space (45–120 cm), social space (129–365 cm), and public space (365–762 cm). Following Hayduk (5), personal space is defined as the area that individuals maintain around themselves where intrusion through others causes discomfort. Intrusions in personal or even intimate space have been related to an increased feeling of threat and increased physiological arousal (6, 7). This is in line with findings that show increased activation of the amygdala for close IPD (8). IPD is therefore a salient feature of social interaction [(9) for an overview]. Importantly, IPDs reflect both avoidance-related and approach-related behavior. A recent study investigated the influence of a fairness manipulation on distance and gaze behavior in Virtual Reality (10). While participants generally avoided unfair agents, the reversed pattern, i.e., approach toward unfair agents, was observed for participants who actively punished unfair agents. This demonstrates the sensitivity of distance measures to different motivational behaviors. Furthermore, IPD is of great interest for the investigation of mental disorders where processing of social information may be affected, like social anxiety disorders and autism spectrum disorders (11, 12).

Social anxiety is characterized by the fear of negative evaluation through others (13). This fear is typically related to social situations, like eating in public, giving a talk, or informal conversations. Highly social-anxious individuals perceive social stimuli as more threatening and this also relates to IPD, where close distances are perceived more threatening than longer distances (11, 14–16). Furthermore, in Virtual Reality paradigms, social anxiety has been related to avoidance behavior such as backward head motion, aversion of eye contact, slow approach and increased distance to virtual agents (14–16). These studies highlight the role of IPD in social interaction and suggest IPD as a target for the investigation of social anxiety. However, so far no studies have investigated the influence of social anxiety for a range of IPDs while measuring subjective experience, physiology, and behavior.

Besides the important role of IPD in social interaction only a small number of studies have systematically investigated the influence of IPD on experience, physiology, and behavior (17). Typically, the stop-distance paradigm has been employed to study IPD and personal space [see (5)]. In this paradigm, the participant approaches an experimenter/confederate and stops as soon as the closeness feels uncomfortable (active stop task). Alternatively, the experimenter/confederate approaches the participant and is stopped by the participant (passive stop task). The stop-distance technique shows high reliability (17) and possesses moderate ecological validity. Furthermore, the stop-distance technique has been successfully applied both in real and in virtual settings (18). However, while the paradigm may be useful to measure personal space itself, there are some limitations when it comes to the study of IPD during social interaction. First, it might be difficult to reach full control over other non-verbal cues, such as eye-gaze and body posture. These cues have been shown to influence social interaction (19) and are directly related to IPD (15). Secondly, even when non-verbal cues are carefully controlled, for example, in a virtual reality paradigm, the absence of other social cues might render the interaction unrealistic. Social interaction is a dynamic process between two or more interaction partners, where all partners respond to social cues elicited by each other (20). Lastly, using the stop-distance technique, it is difficult to sample measures at various distances [but see (17)]. Therefore, data on the effects of IPD is limited to a few sample points and a systematic investigation of the effects of IPD on subjective experience, physiology and behavior has been missing.

The goal of the current study was to address these issues by systematically investigating the influence of IPD on experience, physiology, and behavior in real-time social interaction and to further relate these measures to social anxiety. For that reason, a novel experimental paradigm was implemented in Virtual Reality where participants had to approach virtual agents and engage in minimal social interactions. Crucially, IPD was varied by manipulating the reaction distance (1 to 3.5 m) at which the virtual agents responded to the participants' approach by changing from a passive to a responsive mode, i.e., looking up. This allowed varying IPD in a controlled manner while presenting real-time social interactions, where virtual agents directly responded to participants' approach. Subjective experience of these interactions was assessed *via* ratings of arousal, valence, and realism. Autonomic activity (ECG, EDA) was continuously measured during approach and interaction to test the influence of IPD on physiological arousal. Finally, we evaluated participants' movements once the reaction distance had been reached in order to characterize avoidance behavior.

We hypothesized that participants would rate close IPD in social interaction as more arousing, less pleasant, and less realistic compared to intermediate and remote distances. Furthermore, close distances should elicit increased autonomic activity in terms of skin conductance response (SCR) and changes in heart rate (HR). We also expected to find increased avoidance and reduced approach behavior at close distances. Finally, it was hypothesized that these effects should be modulated as a function of social anxiety, with high social-anxious individuals showing increased sensitivity to close distances compared to low social-anxious individuals.

## Methods

### Participants

Forty healthy adults participated in the present study. Four participants had to be excluded due to technical problems during data acquisition. The remaining 36 participants were healthy students who did not report any mental or neurological disease (mean age = 21.75, sd = 3.03, range 18–34, 18 female). Participants received credit points as compensation. For two participants, distance measures were not recorded and these participants were excluded from the analysis of avoidance behavior. Experimental procedures were in line with the Declaration of Helsinki and the study was approved by the ethics board of the German Society for Psychology (DGPs).

### Questionnaires

Questionnaires were used to assess social anxiety [SPIN (21, 22)], presence [IPQ (23)], state and trait anxiety [STAI (24)], as well as demographic information. Using the median split of the Social Phobia Inventory score (median = 16.5), participants were assigned into two groups: Low social-anxious participants (LSA, SPIN mean = 11.78, sd = 3.95) and high social-anxious participants (HSA, SPIN mean = 24.89, sd = 5.95). **Table 1** depicts comparisons of groups with respect to assessed questionnaires: SPIN, State Trait Anxiety Inventory and the iGroup Presence Questionnaire (Subscales: Spatial Presence, Involvement, Experiences Realism and General item). With respect to the STAI, the trait version was assessed only before the start of the experiment and the state version was assessed before and after the experiment. There was a significant difference in the trait anxiety score as well as in the post experiment state anxiety score, with higher anxiety in the HSA group compared to the LSA group.

**Table 1:** Mean and standard deviations for all obtained questionnaires separately for the HSA and LSA group.

Measure	HSA Mean (sd)	LSA Mean (sd)	t-test p-value
SPIN	24.89 (5.95)	11.78 (3.95)	<.001
Age	21.78 (2.41)	21.72 (3.61)	.957
STAI Trait	43.03 (7.00)	34.73 (6.68)	<.001
STAI State Pre	39.83 (10.19)	34.61 (7.29)	.087
STAI State Post	38.67 (8.38)	32.77 (5.82)	.020
IPQ-G	4.17 (1.65)	4.39 (1.09)	.639
IPQ-SP	2.68 (1.03)	2.58 (0.46)	.711
IPQ-INV	3.88 (0.90)	3.90 (0.90)	.926
IPQ-ER	2.79 (0.84)	2.57 (0.70)	.393

Comparison between groups was done using Welch two sample t-tests. SPIN, Social Phobia Inventory; STAI, State Trait Anxiety Inventory; IPQ, iGroup Presence Questionnaire with subscales; G, General; SP, Spatial Presence; INV, Involvement; ER, Experienced Realism.

### Apparatus

**Figure 1:**
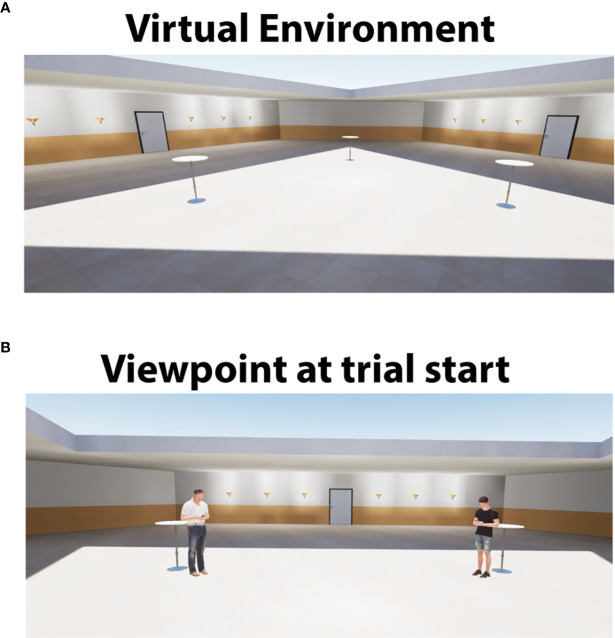
**(A)** Virtual Environment with no virtual agents present. **(B)** Virtual environment at the beginning of a trial with two agents in passive mode. Participants were equidistant from both agents (always 6 m).

The virtual environment was presented *via* head mounted display (HMD, HTC Vive) and participants wore headphones for auditory stimuli. The Virtual environment was created using the Unreal Engine (Version 4.21, Epic Games). The virtual environment was controlled by a scripted experiment paradigm as well as simulation data acquisition established using the VR experiment control software CyberSession (Version 5.8, VTplus, Würzburg, Germany). During the experiment, participants were located in a virtual room with three tables arranged in a triangular pattern in the center of the room (see **Figure 1**). Size of the participants' avatars was always set to 170 cm, so that participants were about the same height as the virtual agents. The body of the avatar of the participants was not displayed. Distance between tables was always six meters. Participants navigated freely through the virtual room by using a gamepad held in the right hand. There were three virtual agents (all male) each with a fixed location at one of the three tables. Agents were either in a passive mode in which they looked at their mobile phone or in a responsive mode in which they looked up from the mobile phone and directed their eye-gaze toward the participant. The transition from passive to responsive mode was triggered when participants reached a specific distance to the agent. These distances were the main experimental manipulation and varied between 1 and 3.5 m in steps of 0.5 m (6 distances in total). For each agent, a pre-recorded audio segment of “*Hello*” was available.

### Measures

Physiological measurements included ECG, EDA, and EMG. For ECG recordings two electrodes were attached to the chest, one at the sternum and one at the left, lower coastal arch. With respect to EMG recordings, each two Ag/AgCl electrodes were positioned on the neck above the left and right *Musculus trapezius*. Reference and ground electrodes were located on the left and right mastoid, respectively. Skin conductance was assessed *via* two electrodes located on the palmar surface of the left hand. All physiological data was recorded using a V-Amp amplifier (BrainProducts, Gilching, Germany) with a sample rate of 1000 Hz. Data analysis of physiological measures was only conducted for ECG and EDA. EMG data was not further analyzed. In order to allow for free movements, participant wore the amplifier in a bag attached to a belt.

As a behavioral measurement, we recorded the distance between participant and each of the agents as a continuous measurement with a sample rate of 90 Hz. The distance was calculated from head of the participant to the heads of the virtual agents.

In order to synchronize data collection from different sources (i.e., physiology and distances) we used Lab Streaming Layer and recorded data with the Lab Streaming Recorder (25).

Furthermore, ratings were assessed in every trial following the interaction with the agent. Ratings were obtained for arousal (“*How high is your emotional arousal?*”), valence (“*How pleasant do you feel?”)*, and realism (“*How natural was the interaction?”)*. All ratings were given on a scale from 0 to 100.

### Procedure

After electrode preparations, participants were introduced into the virtual environment. Initially, there was an exploration phase of 2 min, where participants navigated freely through the virtual room with no agents present. This initial exploration phase was conducted to accustom participants to the virtual environment.

After completion of the exploration phase, the actual experiment was started. There were 36 trials. At the beginning of a trial participants were located at one of the three tables, with two virtual agents standing at the two other tables in front of them (left and right side, see **Figure 1**). Virtual agents were in a passive mode, each staring at a smart phone. There was no agent at the table where the participants were located. After a delay of 1 s, an audio instruction was presented *via* headphones which asked the participant to approach and greet one of the agents (left or right side was balanced across trials). There were 12 trials per agent and the order of agents was pseudorandomized. Initially, navigation was disabled to prevent participants from leaving the starting position before or during the instruction. After the audio instruction, navigation was enabled and participants moved toward one of the agents. At a specific pre-defined distance agents changed from the passive mode to the responsive mode by looking up and fixating the participant (reaction distance). The order of reaction distances over trials was pseudorandomized. In total, there were six trials per reaction distance with two trials per reaction distance per agent. Participants were instructed to greet the agent, as soon as the agent responded to their approach by looking up. The agents then responded by saying “*Hello*” (the agent's response was controlled by the experimenter). Following this interaction, participants were asked to rate arousal, valence, and realism on a scale from 0 to 100 (a score of 100 was indexed as highly arousing, pleasant or realistic). After the ratings, the next trial started. The starting position of the new trial was always the table which had been approached in the previous trial. A trial lasted for about 40 s. There was a break of self-determined length after 18 trials. The total duration of the experiment was about 30 min.

### Data Processing

Data analysis was conducted in Matlab (Mathworks, USA). Preprocessing pipelines were adapted to requirements of the individual measures.

Preprocessing of the ECG data included referencing of the ECG channels, filtering (highpass: 5 Hz, lowpass: 30 Hz, notch: 50 Hz). For HR analysis, R waves were identified using a Matlab implementation of the Pan-Tompkin algorithm (26). Data was segmented into epochs of 6 s following the initial reaction of the agent (i.e., the onset of the agent's change into the responsive mode). HR was calculated for all segments and then exported for statistical analysis.

With respect to EDA data, a lowpass filter with a cut-off of 1 Hz was applied. In analogy to HR processing, EDA data was segmented into epochs related to the initial reaction of the agent (1 s baseline pre onset and 6 s post onset of the change into the responsive mode). Epochs were baseline corrected using the pre onset interval and peak amplitude was identified between 2 and 6 s post onset and exported for statistical analysis.

**Figure 2:**
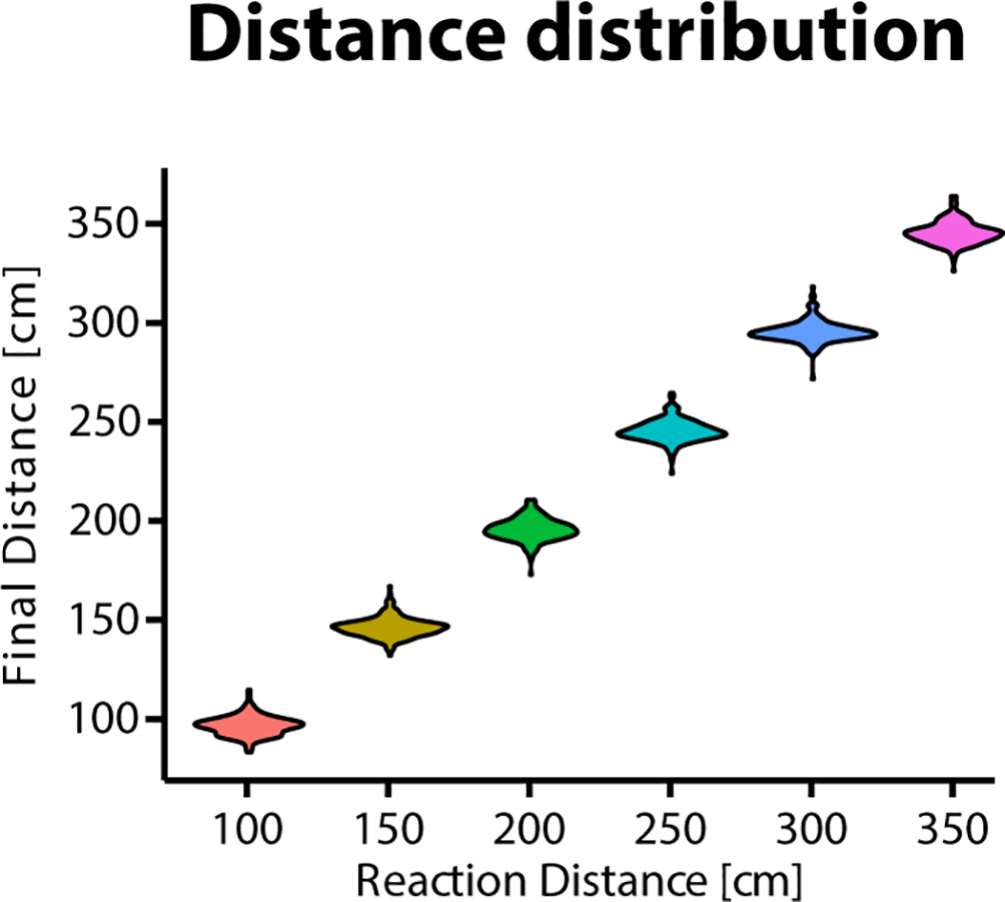
Violin plot depicting the distribution of final distances which were set by the participants after the virtual agents changed from a passive to an active mode.

For behavioral data analysis, the distance between participant and agent was processed in order to extract two measures, approach distance and avoidance distance. With respect to the approach distance, the minimum distance was extracted that participants set to the virtual agents after the agent had changed into the responsive mode. The avoidance distance was then calculated as the maximum distance which participants would establish between themselves and the agent after the final approach distance had been adjusted with the gamepad, which served as a baseline. Importantly, these two distances measured different aspects of movement and distance: the approach distance was mainly determined by participants' movement *via* the gamepad and served as a manipulation check as it allowed to ensure that participants stopped at the reaction distance without restricting movement. **Figure 2** displays the distribution of distances at which participants stopped for each reaction distance. In contrast, the avoidance distance was analyzed as a dependent measure as it is more related to changes in body posture after the general distance had been set with the gamepad.

Finally, ratings (arousal, valence, realism) were averaged across trials for each distance (six trials per distance).

### Statistical Analysis

Statistical analysis was performed using R (27). In order to standardize data for inter-individual differences, the maximal reaction distance of 3.5 m was taken as a reference distance and measures at all other distances were computed in relation to the individual reference. All measures were then analyzed using ANOVAs with *Reaction Distance* as within-subject factor (five levels: 1, 1.5, 2, 2.5, 3 m; all in relation to the reference at 3.5 m) and *Social anxiety* as between-subject factor (two levels: HSA and LSA). Violations of sphericity were corrected using the Greenhouse-Geisser method (28). Significant effects were followed-up with *post hoc* t-tests with a correction for multiple comparisons according to Holm (29). As we hypothesized to find increased effects in the HSA group compared to the LSA group, one-sided t-tests were used when the assessing group differences for particular distances. Significance tests were conducted with α = 0.05.

## Results

### Ratings

#### Arousal

**Figure 3:**
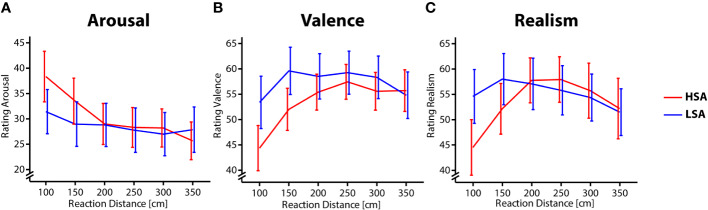
Ratings for different Reaction Distances and Social Anxiety. High social-anxious participants are shown in red and low social-anxious participants are shown in blue. Error bars reflect the standard error of the mean. **(A)** Arousal ratings, **(B)** Valence ratings, and **(C)** Realism ratings.

A mixed ANOVA with *Reaction distance* as a within-subject factor and *Social Anxiety* as between-subject factor revealed a main effect of *Social Anxiety, F*(1,34) = 5.25, *p* =.028, *η_p_^2^* = 0.134, and a main effect of *Reaction Distance, F*(4,136) = 11.86, *p* < .001, *η_p_^2^* = 0.259 (*ε* = 0.49), as well as a trend for the interaction between *Social Anxiety* and *Reaction Distance, F*(4,136) = *2.82*, *p* =.068, *η_p_^2^* = 0.077 (*ε* = 0.49). Arousal ratings were increased in the HSA group compared to the LSA group. Social interactions at a distance of 1 and 1.5 m were rated as more arousing than longer distances greater 2 m. The interaction effect, although only trending, suggested increased Arousal at short distances in the HSA group compared to the LSA group [1 m: *t*(24.54) = 2.38, *p* =.051, *d* = 0.793; 1.5 m: *t*(22.87) = 2.59, *p* =.041, *d* = 0.862; other distances *p* > .10]. In summary, ratings of arousal differed as a function of both social anxiety and reaction distance (**Figure 3A**).

#### Valence

Valence ratings revealed a main effect of effect of *Social Anxiety, F*(1,34) = 4.63, *p* =.039, *η_p_^2^* = 0.12, and a main effect of *Reaction Distance F*(4,136) = 11.79, *p* < .001, *η_p_^2^* = 0.258 (*ε* = 0.58). There was no interaction of both factors, *F*(4,136) = 2.18, *p* =.115. Participants in the HSA group rated the interactions as less pleasant compared to the LSA group and interactions at 1 m distance were rated as less pleasant compared to longer distances [1 m vs. 1.5 m: *t*(35) = −4.67, *p* < .001, *d* = 0.777; 1 m vs. 2 m: *t*(35) = −4.23, *p* =.001, *d* = 0.705; 1 m vs. 2.5 m: *t*(35) = −4.30, *p* =.001, *d* = 0.694; 1 m vs. 3 m: *t*(35) = −4.67, *p* =.001, *d* = 0.716]. These data demonstrate that pleasantness of social interaction in VR was affected both by distance and social anxiety (**Figure 3B**).

#### Realism

With respect to the ratings of the realism of an interaction, there was a main effect of *Reaction Distance, F*(4,136) = 6.13, *p* =.001, *η_p_^2^* = 0.153 (*ε* = 0.62), and an interaction effect between *Social Anxiety* and *Reaction Distance, F*(4,136) = 4.56, *p* =.008, *η_p_^2^* = 0.118 (*ε* = 0.62). *Post hoc* t-test revealed that the interactions at a short distance were rated as less realistic compared to longer distances in the HSA group (1m vs. 1.5m: *t*(17) = −3.19, *p* =.036, *d* = 0.751; 1 m vs. 2 m: *t*(17) = −3.24, *p* =.036, *d* = 0.764; 1 m vs. 2.5 m: *t*(35) = −3.27, *p* =.036, *d* = 0.771; 1 m vs. 3 m: *t*(35) = −2.96, *p* =.044, *d* = 0.697) but not in the LSA group (all *p* > .5). Therefore, social interactions at a short distance were rated as less realistic compared to longer distances but this effect was only present in high social-anxious participants (**Figure 3C**).

### Physiological Parameters

**Figure 4:**
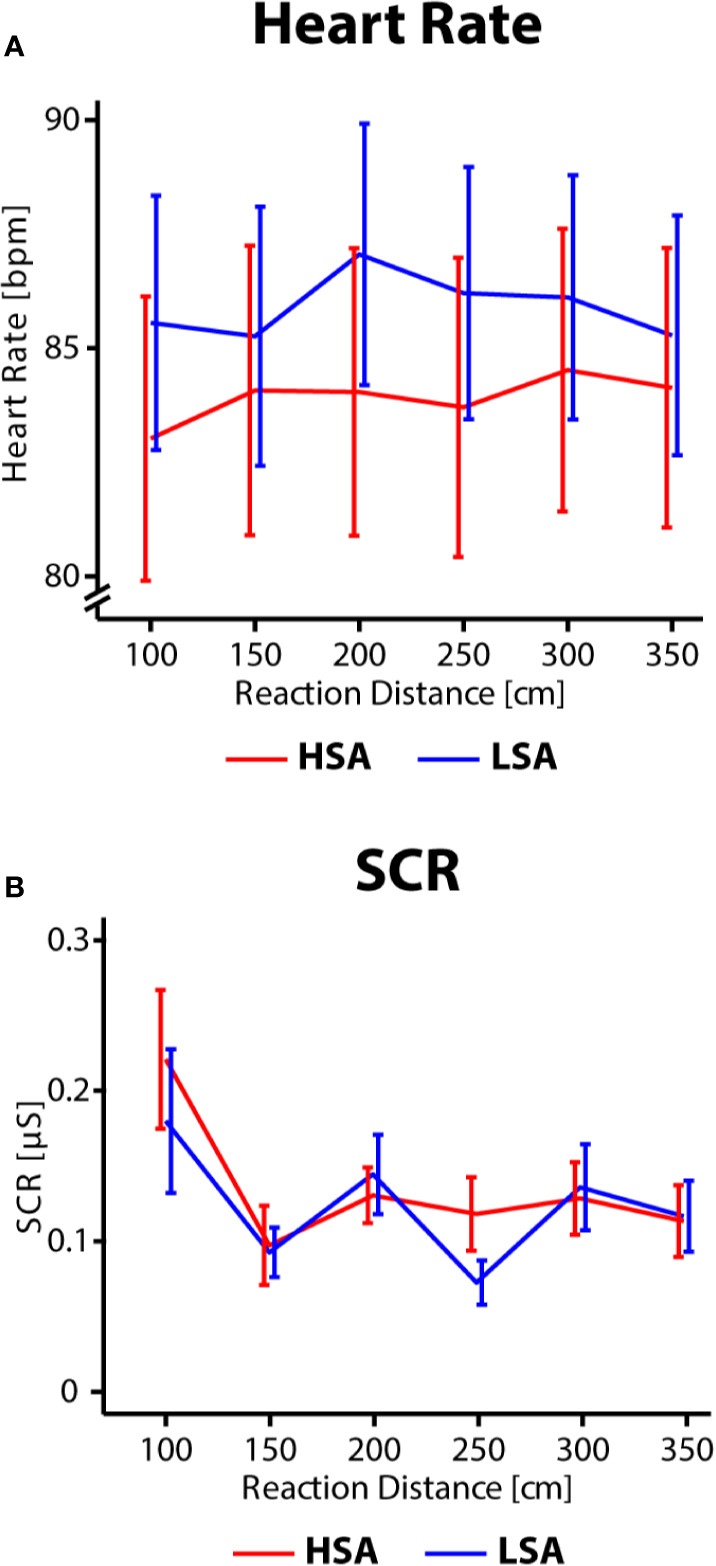
**(A)** Heart rate in beats per minute (bpm). **(B)** SCR in microSiemens [μS]. Data are shown for different Reaction Distances and Social Anxiety. High social-anxious participants are shown in red and low social-anxious participants are shown in blue. Error bars reflect the standard error of the mean.

As physiological variables, HR (in the six seconds following the agents initial reaction) and SCR (elicited by the initial reaction of the agent) was analyzed. With respect to HR there was no significant main effect or interaction (all *F* < 1, see **Figure 4A**). With respect to SCR, the ANOVA revealed a significant main effect of *Reaction Distance, F*(4,136) = 9.54, *p* < .001, *η_p_^2^* = 0.219 (*ε* = 0.55). There was no main effect of *Social Anxiety* and no interaction effect (all *F* < 1). *Post hoc* t-test revealed that an agent's reaction at a short distance of 1 m elicited an increased SCR compared to longer distances [1 m vs. 1.5 m: *t*(35) = 4.26, *p* =.001, *d* = 0.711; 1 m vs. 2.5 m: *t*(35) = 3.86, *p* =.004, *d* = 0.645; 1 m vs. 3 m: *t*(35) = 273.86, *p* =.059, *d* = 0.456; all other distances: *p* > .1]. These data show that physiological arousal, as indexed by SCR, was sensitive to reaction distance in social interactions (**Figure 4B**).

### Behavior

**Figure 5:**
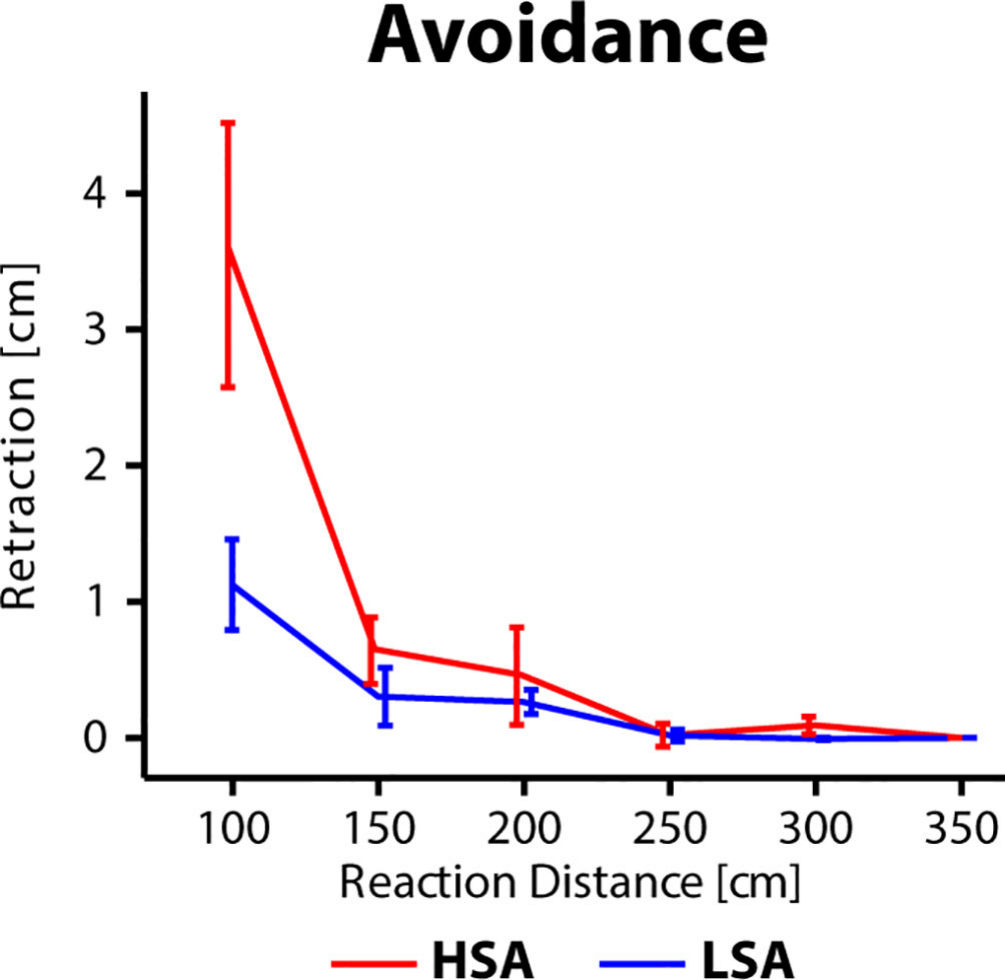
Distance in cm by which participants retracted from the virtual agent after the final position was set with the gamepad. Data shown for different Reaction Distances and Social Anxiety. High social-anxious participants are shown in red and low social-anxious participants are shown in blue. Error bars reflect the standard error of the mean.

The distance by which participants retracted from a virtual agent was analyzed as a behavioral measure of avoidance. The ANOVA revealed a main effect of *Social Anxiety*, *F*(1,32) = 4.91, *p* =.034, *η_p_^2^* = 0.154, a main effect of *Reaction Distance, F*(4,128) = 13.97, *p* =.001, *η_p_^2^* = 0.331 (*ε* = 0.35), and an interaction effect between *Social Anxiety* and *Reaction Distance, F*(4,136) = 3.84, *p* =.044, *η_p_^2^* = 0.13 (*ε* = 0.35). *Post hoc* t-tests revealed that there was a trend toward increase of avoidance in the HSA compared to the LSA group at a distance of 1 m, *t*(19.65) = 2.48, *p* =.056, *d* = 0.787, other distances *p* > .4. In summary, there was increased retraction away from the virtual agent at a short interaction distance in the HSA group compared to the LSA group (see **Figure 5**).

## Discussion

The present study varied the distance at which participants engage in social interaction with virtual agents. This was achieved by manipulating the distance at which the virtual agents reacted to the participants' approach by switching from a passive to an active mode, i.e., by looking up and focusing on the participant. The results show that physical distance during social interaction affects subjective experience as well as physiological parameters and behavioral avoidance. Social interactions at a close physical distance of one meter were rated as more arousing, less pleasant, and less realistic compared to distances above two meters and elicited an increased physiological response as reflected in the SCR. Finally, participants also showed increased avoidance at a close distance compared to longer distances. Importantly, the subjective experience with respect to arousal and realism as well as the behavioral avoidance also differed as a function of social anxiety. In detail, high social-anxious participants rated interactions at close distances as less realistic and more arousing compared to low social-anxious participants. Furthermore, high social-anxious participants displayed more avoidance behavior at the closest distance compared to low social-anxious participants.

The increased arousal ratings and reduced pleasantness ratings are in line with the existing literature that show increased threat perception elicited by close IPDs within the personal space (6, 7). The effect of IPD on arousal was further modulated by social anxiety suggesting that social cues and especially close distances are perceived as even more threatening for persons with high social anxiety (11, 14–16). However, this interaction between distance and social anxiety was not reflected in the physiological parameters. While there was a general effect of IPD on physiological arousal, as indexed by SCR [see Ref. (6) for an evaluation of IPD using startle probes], this effect was similar for high and low social-anxious participants. This was unexpected, as one might predict that the increased perception of threat might also lead to an increased physiological response. Previous studies, however, have shown that physiological responses are often similar between high and low social-anxious participants (15, 30). These and our findings suggests that emotion processing in social anxiety might be related to the interpretation of physiological states rather than actual physiological responding (30).

Importantly, we found an effect of IPD not only for arousal and valence but also for realism. To our knowledge, the present study is the first to assess realism ratings with respect to IPD in VR. Crucially, realism of social interactions was rated differently between persons with high and low social anxiety scores. LSA participants did not differentiate between IPDs with respect to realism, HSA participants, however, rated social interactions at close distances as less realistic. This suggests that high social-anxious persons differ in their beliefs about “normal” social interaction from low social-anxious persons. One could speculate that this might be the case because HSA persons are more likely to evaluate social interactions on the basis of their own subjective experience and not on the basis of social cues provided by their interaction partners (13).

The rating data should also be discussed with respect to the Uncanny Valley Hypothesis [UVH (31)]. The UVH states that humanlike characters who are close to real humans but do not completely resemble them will induce a negative affective state. It is possible that the relation between distance and pleasantness was modulated by the uncanny valley effect, as anthropomorphic features may be more prominent at closer distances. Note, however, that results with respect to the UVH are mixed and even contradictory results, i.e., increased pleasantness related to increasing human likeness, have been reported (32). Furthermore, the present study showed not only reduced pleasantness but also reduced realism ratings for close distances (at least in HSA participants). Therefore, it is unlikely that the present effects were solely driven by the Uncanny Valley effect. However, this should be further investigated in future studies where human likeness is explicitly manipulated.

Finally, we observed increased avoidance in HSA participants related to IPD that was reflected in retraction from the virtual agents. This finding is in line with previous measures of avoidance behavior such as reduced eye-contact, backward head movements or speed of approach (10, 14, 15, 33). In a previous study by Wieser et al. (15), avoidance behavior was related to social anxiety, but there was no modulation of avoidance with respect to IPD. Note, however, that in the study by Wieser et al., the agent moved toward the participant while the participant remained stationary. Therefore, one explanation might be that in the active approach toward the agent might increase the salience of distance and thereby result in increased avoidance behavior with respect to IPD. This should be addressed in future experiments. Furthermore, these studies should include measurements of eye gaze as previous studies have highlighted the relation of distance and gaze direction (3, 10, 34).

The present experiment highlights Virtual Reality as a technique for the study of social interaction. High experimental control while maintaining naturalistic settings are key advantages of VR. This is especially relevant for the investigation of IPD because of the limitations of presenting controlled social interactions. Our results as well as previous work show that real and virtual social stimuli elicit similar responses (35, 36). Here, we demonstrate that a paradigm in Virtual Reality is sensitive to even small manipulations of distance as well as to inter-individual variation in social anxiety. These advantages of social interactions in VR may also be of interest for therapeutic use. It has been demonstrated that VR exposure therapy can be successfully used with patients suffering from social anxiety (37). On the basis of our results, we suggest to implement distance manipulations as a tool in virtual exposure therapy.

It has to be acknowledged, however, that it is quite challenging to provide highly realistic social interactions in VR. In the present experiment, social interactions were defined as a short greeting between participant and virtual agent, where the response of the virtual agent was controlled by the experimenter. Technological advances might help in future studies to test more elaborate interactions including dialogues with a virtual agent. Another limitation of the present experiment is that the individually preferred IPD was not assessed. Again, this should be addressed in future studies by adding a session with the stop-distance technique to the experiment and relating the assessed distances to the preferred distance. This should increase the sensitivity to effects of IPD.

The analysis of social anxiety on the basis of a median split combined with a relatively small sample size brings some limitations with respect to statistical power. It should be noted, however, that the median in the present sample (16.5) was only 2.5 point below a cut-off score of 19 that has been suggested to distinguish between social phobia subjects and controls (38). Therefore, the present group analysis might be useful for evaluating the role of physical distance with respect to clinical applications. Nevertheless, the present study should be seen as a starting point for future investigations with increased sample size.

Summarizing, we measured effects of IPD and social anxiety on subjective experience, physiology, and behavior during real-time social interaction in Virtual Reality. Our results show increased arousal, reduced valence and, for the first time, reduced realism for close IPDs. The effects on arousal and realism appear to be amplified in high social-anxious participants in comparison to low social-anxious participants. IPD also affected SCR in both groups. Finally, we observed increased avoidance behavior for close distances in high social-anxious participants. In total, these results suggest Virtual Reality is able to induce relevant verbal and nonverbal emotional responses in virtual social settings and thus is a useful tool in studying social interaction and developing interventions for social training purposes or psychotherapy.

## Data Availability Statement

The raw data supporting the conclusions of this article will be made available by the authors, without undue reservation.

## Ethics Statement

The study involving human participants was reviewed and approved by Ethics Committee of the Deutsche Gesellschaft für Psychologie (DGPs). The patients/participants provided their written informed consent to participate in this study.

## Author Contributions

LK, MP, and AM designed research. LK programmed the experiment, supervised data acquisition, and analyzed data. BL and MM supervised the virtual environment creation and experiment paradigm programming. LK, MP, and AM wrote the paper. BL and MM commented the paper.

## Funding

This study was supported by the German Federal Ministry of Education and Research project “OPTAPEB” (FKZ: 16SV7839K; Bundesministerium für Bildung, Wissenschaft, Forschung und Technologie).

## Conflict of Interest

AM and MM are shareholders of a commercial company (VTplus GmbH) that develops virtual environment research systems for empirical studies in the field of psychology, psychiatry, and psychotherapy. MM is an executive officer and BL is an employee of the same company.

The remaining authors declare that the research was conducted in the absence of any commercial or financial relationships that could be construed as a potential conflict of interest.
